# Muscle cramps as disorders of impaired termination of contraction: An integrated neurophysiological framework

**DOI:** 10.14814/phy2.71001

**Published:** 2026-07-03

**Authors:** Akira Takahashi

**Affiliations:** ^1^ Dialysis Center Tesseikai Neurosurgical Hospital Shijonawate Japan

**Keywords:** calcium handling, electromyography, metabolic stress, motor unit activity, neural disinhibition, skeletal muscle relaxation

## Abstract

Muscle cramps are common neuromuscular phenomena observed across diverse clinical and physiological settings, including hemodialysis and exercise. Although altered motor neuron excitability is considered a central mechanism, the physiological processes underlying the persistence and termination of cramp activity remain incompletely understood. This narrative review integrates neurophysiological, metabolic, and peripheral physiological evidence to propose an integrated framework for muscle cramp persistence, with particular emphasis on sustained motor unit activity, inhibitory control, calcium handling, and energetically supported relaxation processes. Current evidence suggests that sustained motor unit activity and altered spinal inhibitory control represent key mechanisms underlying muscle cramps. In addition, metabolically stressed conditions, altered calcium handling, impaired energetic support for ATP‐dependent relaxation processes, and altered cross‐bridge kinetics may contribute to inefficient termination of contraction. These interacting neural, metabolic, and peripheral physiological factors may help explain the persistence and variability of cramp activity across different clinical contexts. Muscle cramps may be better understood not simply as disorders of excessive activation, but as conditions involving impaired termination of contraction arising from interacting neurophysiological and metabolic mechanisms. This integrated framework may provide a useful conceptual and physiological basis for future mechanistic and translational investigation.

## Graphical abstract

This graphical abstract illustrates an integrated neurophysiological framework in which muscle cramps arise from interactions among chemical, metabolic, and neural mechanisms that impair the termination of muscle contraction. This framework provides a unifying perspective for understanding muscle cramps across hemodialysis, exercise, aging, and other clinical or physiological conditions.

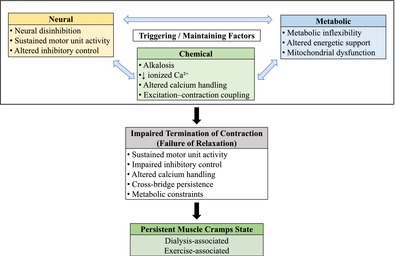

## 1 INTRODUCTION

Muscle cramps are a common musculoskeletal symptom observed across diverse clinical and physiological conditions, including aging, neuromuscular disorders, pregnancy, exercise, and hemodialysis (Bordoni et al., 2025; Canzanello & Burkart, 1992). Although typically transient, cramps can cause severe pain, impaired function, sleep disturbance, and interruption of ongoing physical activity or medical treatment. Despite their high prevalence, the precise mechanisms underlying muscle cramps remain incompletely understood, and it remains unclear to what extent different forms of cramping share common pathophysiological pathways.

Current prevailing theories generally emphasize altered motor unit excitability and abnormal neuromuscular activation as central mechanisms underlying muscle cramps. In particular, exercise‐associated muscle cramps have frequently been explained by an imbalance between excitatory input from muscle spindles and inhibitory input from Golgi tendon organs, leading to sustained alpha motor neuron firing (Miller & Burne, 2014; Schwellnus et al., 1997). Similarly, dialysis‐associated muscle cramps have been associated with hemodynamic instability (Kaplan et al., 1992; Santoro et al., 1998), electrolyte imbalance (Kasserra et al., 1994; Wagner et al., 1983), and neuromuscular hyperexcitability. However, while these mechanisms may help explain the initiation of muscle contraction, they do not fully explain why muscle contraction, once initiated, may become prolonged and difficult to terminate in certain situations. To better understand the persistence of muscle cramps, it may also be important to consider the physiological processes involved in muscle relaxation itself. Skeletal muscle relaxation depends on both the termination of motor neuron activation and the ATP‐dependent reuptake of calcium into the sarcoplasmic reticulum, processes that may be impaired under pathological or metabolically stressed conditions.

In hemodialysis, ultrafiltration‐induced alkalosis (Garella et al., 1975; Haskins et al., 2006), altered calcium handling (Nakamaru & Schwartz, 1972; Pedersen, 1972), metabolic disturbances (Sakurauchi et al., 1998; Takahashi, 2021), and changes in inhibitory neural control (Schwellnus et al., 1997) have all been implicated in muscle cramp development. These observations raise the possibility that mechanisms responsible for terminating muscle contraction may become impaired under physiologically stressed conditions.

Based on these considerations, it may be useful to conceptualize dialysis‐associated muscle cramps not only as disorders of excessive motor unit activation but also as conditions involving impaired relaxation processes (“failure of relaxation”). This perspective shifts the focus from the initiation of contraction to the impairment of relaxation processes, particularly ATP‐dependent calcium reuptake (Block et al., 1988; Dulhunty & Gage, 1988) and neural inhibition. Such a reframing may provide a more comprehensive explanation for the prolonged and treatment‐resistant nature of cramps observed during dialysis.

Similar physiological stresses may occur in exercise‐associated muscle cramps, suggesting that common mechanisms may contribute across different cramp conditions.

This narrative review integrates neurophysiological, metabolic, and biochemical evidence to propose an integrated framework of muscle cramps centered on impaired termination of contraction (“failure of relaxation”).

## 2 CLASSICAL MECHANISMS OF DIALYSIS‐ASSOCIATED MUSCLE CRAMPS

Muscle cramps during hemodialysis have traditionally been attributed to a combination of hemodynamic, biochemical, electrolyte, acid–base, and metabolic factors. Previous studies have described intradialytic hypotension, rapid ultrafiltration, changes in extracellular volume, electrolyte disturbances, acid–base shifts, and carnitine deficiency as potential contributors to dialysis‐associated muscle cramps (Bordoni et al., 2025; Borum & Taggart, 1986; Canzanello & Burkart, 1992; Evans, 2003; Hatanaka et al., 2019; Kaplan et al., 1992; Kasserra et al., 1994; Noble, 2022; Sakurauchi et al., 1998; Santoro et al., 1998; Takahashi, 2021; Wagner et al., 1983; Wanner & Hörl, 1988). However, these mechanisms are often discussed separately, and none appear sufficient to explain all clinical presentations of cramping. Therefore, they may be better understood as interacting contributors rather than isolated causes.

### 2.1 Hemodynamic factors: Fluid removal and hypotension

Excessive ultrafiltration may reduce skeletal muscle perfusion and increase susceptibility to cramping (Kaplan et al., 1992; Santoro et al., 1998). However, hypotension alone does not explain all cramp episodes, suggesting that additional mechanisms are involved.

### 2.2 Electrolyte and acid–base disturbances

Electrolyte and acid–base disturbances (Kirkland et al., 2018; Liguori et al., 2024; Maughan & Shirreffs, 2019; Souza et al., 2023), particularly contraction alkalosis (Garella et al., 1975; Takahashi, 2021), may increase neuromuscular excitability through reductions in ionized calcium, which may lower the activation threshold of excitable membranes, as well as through alterations in calcium handling (Nakamaru & Schwartz, 1972; Pedersen, 1972). However, these factors alone do not fully explain cramp persistence.

### 2.3 Carnitine deficiency and impaired energy metabolism

Carnitine deficiency (Borum & Taggart, 1986; Evans, 2003; Hatanaka et al., 2019) and impaired mitochondrial metabolism may reduce energetic support for ATP‐dependent processes involved in muscle relaxation (Noble, 2022). Nevertheless, clinical responses to carnitine supplementation remain variable (Sakurauchi et al., 1998; Wanner & Hörl, 1988), suggesting that these mechanisms are contributory rather than sufficient explanations.

### 2.4 Limitations of the classical framework

While hemodynamic instability, electrolyte disturbances, and impaired energy metabolism each contribute to the development of muscle cramps, these mechanisms are typically considered in isolation. As a result, the classical framework does not adequately explain how these factors interact to produce sustained muscle contraction.

Importantly, most traditional models primarily focus on mechanisms responsible for initiating contraction. In contrast, relatively little emphasis has been placed on the processes required for muscle relaxation, which are both energy‐dependent and tightly regulated by neural and biochemical factors (Nelson & Churilla, 2016).

These limitations highlight the need for a more integrative approach that accounts not only for the initiation of muscle contraction but also for the failure of muscle relaxation.

## 3 A NEW FRAMEWORK: “FAILURE OF RELAXATION”

Skeletal muscle relaxation is a multifactorial process involving termination of motor neuron activation, calcium reuptake into the sarcoplasmic reticulum, and dissociation of actin–myosin cross‐bridges. Disturbances in any of these processes may contribute to delayed relaxation under pathological or metabolically stressed conditions. Although classical mechanisms provide important insights into the development of dialysis‐associated muscle cramps, they do not fully explain why muscle contraction, once initiated, becomes sustained and difficult to terminate. This limitation suggests that muscle cramps should be reconsidered not only as disorders of excessive motor unit activation but also as disorders of impaired relaxation.

Muscle relaxation requires coordinated regulation of ionic balance, energy metabolism, and neural control. Contraction is triggered by calcium release from the sarcoplasmic reticulum (Endo et al., 1970), whereas relaxation depends on ATP‐dependent calcium reuptake via sarcoplasmic reticulum Ca^2+^‐ATPase (SERCA) (Ebashi, 1960; Hasselbach & Makinose, 1961; MacLennan, 1970). Disturbances that enhance calcium release, impair ATP‐dependent processes, or reduce inhibitory neural control may therefore contribute to impaired termination of contraction (Gehlert et al., 2015; Wakabayashi, 2015).

Importantly, relaxation slowing cannot always be explained solely by impaired calcium removal. Westerblad et al. demonstrated that relaxation may be slowed despite relatively preserved calcium uptake capacity, suggesting that altered cross‐bridge kinetics and other mechanisms may also contribute (Westerblad et al., 1997). Accordingly, impaired relaxation is viewed here as a multifactorial phenomenon involving neural, metabolic, calcium‐handling, and cross‐bridge mechanisms rather than impaired SERCA function alone. The relative contribution of these mechanisms may differ according to species, muscle type, and physiological context.

Based on this perspective, muscle cramps are proposed to arise from the interaction of three interrelated layers: (1) chemical factors that increase neuromuscular excitability, (2) metabolic factors that reduce the efficiency of ATP‐dependent relaxation processes, and (3) neural factors that diminish inhibitory control. Together, these factors may converge to produce a state of “failure of relaxation,” in which muscle contraction cannot be appropriately terminated.

### 3.1 Chemical layer: Alkalosis and calcium dynamics

Metabolic alkalosis may alter neuromuscular excitability through changes in extracellular ionized calcium availability. Alkalosis increases the binding of calcium ions to serum albumin, thereby reducing the concentration of ionized calcium in extracellular fluid (Nakamaru & Schwartz, 1972; Pedersen, 1972). Reduced extracellular ionized calcium may lower the activation threshold of excitable membranes by altering surface charge screening and sodium channel availability, potentially increasing susceptibility to neuromuscular activation. In addition, alkalosis‐related changes in intracellular and extracellular pH may influence calcium handling (Nakamaru & Schwartz, 1972) and excitation–contraction coupling (Achike et al., 1997; Spriet et al., 1986).

Experimental and cellular studies suggest that pH changes can modulate ryanodine receptor (RyR) function in a non‐linear manner, although the effects of alkalinization above physiological pH may be relatively modest (Laver et al., 2000).

The effects of dialysis‐related acid–base disturbances on skeletal muscle are likely physiologically complex and may vary according to local metabolic conditions.

### 3.2 Metabolic layer: Energetic support and ATP‐dependent relaxation

Muscle relaxation is an energy‐dependent process requiring ATP for calcium uptake into the sarcoplasmic reticulum and dissociation of actin–myosin cross‐bridges (Ebashi, 1960; MacLennan, 1970; Noble, 2022). Accordingly, disturbances in energy metabolism may influence the efficiency of relaxation processes, particularly under conditions of repeated activation, reduced perfusion, or increased metabolic demand.

Carnitine plays an important role in mitochondrial fatty acid transport and metabolic flexibility, enabling shifts between substrate utilization pathways according to energetic demand (Virmani & Cirulli, 2022). Impaired mitochondrial substrate utilization may therefore alter energetic support for ATP‐dependent cellular processes involved in muscle relaxation.

Carnitine deficiency, mitochondrial dysfunction, and loss of water‐soluble vitamins have all been discussed as potential contributors to altered energy metabolism in patients undergoing hemodialysis (Borum & Taggart, 1986; Evans, 2003; Hatanaka et al., 2019; Noble, 2022; Sakurauchi et al., 1998; Takahashi, 2021; Wanner & Hörl, 1988). Importantly, impaired energy metabolism in this framework refers to altered energetic support for relaxation processes rather than severe depletion of intracellular ATP stores.

Delayed relaxation may involve both altered calcium handling and cross‐bridge kinetics (Westerblad et al., 1997).

### 3.3 Neural layer: Disinhibition and sustained motor unit activity

Among the proposed mechanisms of muscle cramps, sustained motor unit activity and altered neural excitability currently represent some of the most strongly supported explanations, particularly in exercise‐associated muscle cramps (EAMCs) and other conditions characterized by abnormal motor neuron firing (Curtis, 1969; Schwellnus et al., 1997). Electromyographic (EMG) studies have demonstrated that cramp episodes are associated with high‐frequency and sustained motor unit discharge, markedly exceeding the firing rates observed during normal voluntary contraction (Khan & Burne, 2007; Minetto et al., 2013). These findings support the concept that muscle cramps involve abnormal maintenance of neural activation rather than isolated abnormalities within muscle tissue itself.

Current neurophysiological models suggest that muscle cramps may arise from an imbalance between excitatory and inhibitory inputs to alpha motor neurons. The “neuromuscular control theory,” largely developed by Schwellnus and colleagues, proposes that muscle fatigue and metabolically stressed conditions increase excitatory input from muscle spindles while reducing inhibitory input from Golgi tendon organs, resulting in sustained alpha motor neuron firing and abnormal spinal reflex activity (Schwellnus, 2009; Schwellnus et al., 2011). In this context, inhibitory neurotransmitter systems involving γ‐aminobutyric acid (GABA) and glycine may also contribute to modulation of motor neuron excitability within the spinal cord (Eccles et al., 1962; Eliav et al., 2001).

Additional experimental evidence suggests that motor neurons may exhibit bistable firing behavior under certain physiological conditions. Baldissera et al. proposed that once activated, motor neurons may continue firing autonomously unless sufficient inhibitory input is provided (Baldissera et al., 1994). This concept may help explain the persistence and self‐sustaining nature of cramp activity.

Furthermore, studies using peripheral nerve block techniques have demonstrated the importance of spinal circuitry in the maintenance of muscle cramps. Minetto et al. showed that experimentally induced cramps terminated rapidly under peripheral nerve block conditions, whereas cramp activity persisted when spinal connections remained intact (Minetto et al., 2011). These findings support the concept that sustained cramp activity depends not only on peripheral muscle factors but also on ongoing central or spinal neural drive.

The effectiveness of muscle stretching in relieving cramps may also support this neurophysiological framework. Recent evidence suggests that muscle spindle behavior may be influenced not only by muscle length but also by post‐contraction changes in spindle sensitivity and local mechanical factors. Wilson et al. (1995) reported increased resting spindle afferent discharge following voluntary contractions, whereas Torell and Dimitriou (2024) demonstrated that local muscle pressure can directly increase spindle activity. These observations raise the possibility that repeated contractions and elevated intramuscular pressure may contribute to sustained excitatory afferent feedback during cramp activity. Conversely, stretching may help relieve cramps not only through Golgi tendon organ‐mediated inhibition but also by reducing local muscle pressure and normalizing abnormal spindle‐related input. Stretching activates Golgi tendon organs and increases inhibitory input to alpha motor neurons, thereby interrupting sustained motor neuron discharge and facilitating termination of contraction (Schwellnus, 2009). In addition, elongation of the affected muscle may mechanically reduce actin–myosin overlap, alter force–length relationships, and reduce muscle spindle discharge, thereby further decreasing excitatory drive to alpha motor neurons. These combined neural and mechanical effects may contribute to the rapid relief commonly observed with stretching during cramp episodes.

Current neurophysiological models suggest that altered afferent feedback during fatigue or metabolic stress may shift the balance between excitatory and inhibitory inputs to alpha motor neurons (Hutton & Nelson, 1986; Minetto et al., 2011; Nelson & Churilla, 2016; Nelson & Hutton, 1985). Under such conditions, inhibitory regulation of alpha motor neuron activity may become insufficient to appropriately terminate sustained motor unit firing.

Inhibitory spinal interneurons mediated by γ‐aminobutyric acid (GABA) and glycine normally contribute to suppression and termination of excessive motor neuron activity. Fatigue‐related alterations in afferent feedback may reduce the effectiveness of these inhibitory pathways, resulting in insufficient suppression of sustained motor unit activity.

These mechanisms may differ depending on the clinical context. For example, chronic nerve compression may contribute to persistent spinal excitability in some pathological conditions, whereas exercise‐associated cramps may involve transient fatigue‐related alterations in spinal reflex balance. Nevertheless, both situations may converge on sustained motor unit activity and impaired termination of contraction.

At the same time, neural mechanisms may interact with metabolic and peripheral physiological factors. Altered calcium handling, reduced metabolic flexibility, impaired perfusion, or metabolic stress may influence the susceptibility of muscle to sustained contraction once abnormal motor unit activity has been initiated. Therefore, the proposed framework considers sustained neural activation as a key initiating and maintaining mechanism, while recognizing that additional peripheral factors may modulate the persistence, severity, or termination of muscle cramps.

### 3.4 Integration: Convergence toward “failure of relaxation”

Importantly, none of the mechanisms described above should be interpreted as a singular explanation for muscle cramps. Rather, current evidence suggests that neural activation, calcium handling, metabolic state, and cross‐bridge regulation interact to influence the persistence and termination of contraction under different physiological conditions.

Among these mechanisms, sustained motor unit activity and altered neural excitability currently represent some of the most strongly supported explanations for muscle cramps. However, neural activation alone may not fully explain why cramp activity becomes prolonged, self‐sustaining, or difficult to terminate under certain physiological and pathological conditions. Additional factors involving calcium handling, metabolic state, perfusion, and cross‐bridge regulation may influence the persistence and severity of cramp activity once abnormal motor neuron firing has been initiated.

Within this context, the present framework proposes that effective muscle relaxation depends on coordinated termination of motor neuron activation, restoration of ionic balance, ATP‐dependent calcium reuptake, and dissociation of actin–myosin cross‐bridges. Disturbances affecting any of these processes may contribute to impaired termination of contraction.

Importantly, the proposed “failure of relaxation” framework should not be interpreted as a singular mechanistic explanation centered solely on impaired SERCA function or ATP depletion. Rather, it is intended as an integrative physiological concept describing conditions in which normal termination of contraction becomes inefficient or incomplete because of interacting neural and peripheral factors.

This integrated perspective may help explain several clinical features of muscle cramps, including sustained cramp activity, variability in symptom severity, and differences in therapeutic response among patients. It may also help reconcile apparently conflicting observations regarding neural activation, calcium handling, metabolic stress, and muscle fatigue within a unified physiological framework.

**FIGURE 1:**
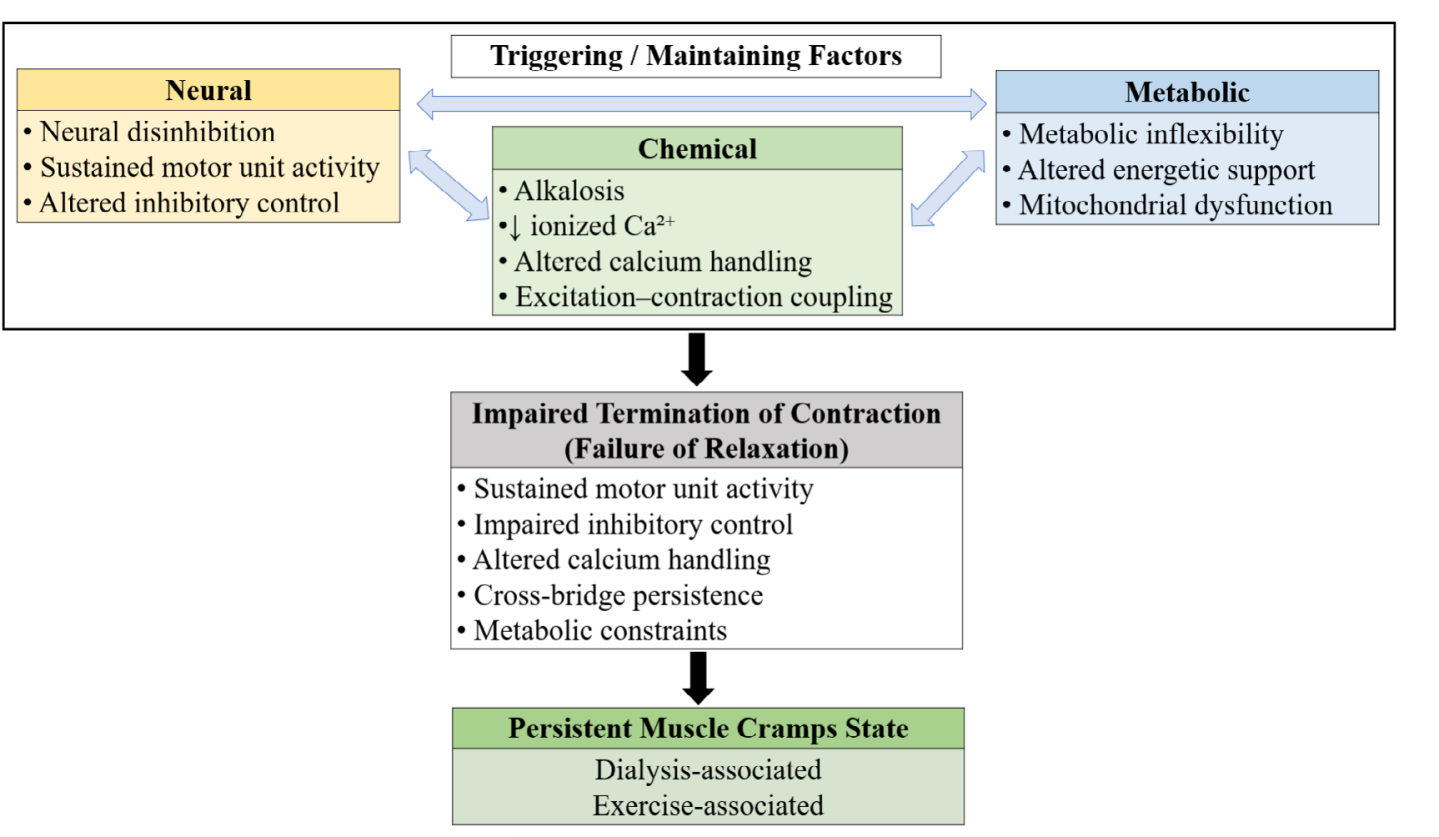
Integrated framework of muscle cramps centered on impaired termination of contraction (“failure of relaxation”). The model illustrates how neural disinhibition, altered calcium handling, and metabolic stress may interact to impair termination of muscle contraction. Sustained motor unit activity is proposed as a central mechanism, while altered energetic support, excitation–contraction coupling, and cross‐bridge persistence may contribute to cramp persistence under metabolically stressed conditions.

Accordingly, muscle cramps may be better understood not simply as disorders of excessive motor unit activation, but as disorders involving impaired termination of contraction arising from the convergence of neural disinhibition, altered calcium regulation, metabolic constraints, and impaired relaxation processes (Figure 1).

## 4 CLINICAL IMPLICATIONS

The proposed framework of muscle cramps as involving impaired termination of contraction may provide a useful physiological perspective for multidimensional clinical management. Rather than focusing solely on suppression of muscle contraction itself, this framework highlights the potential importance of neural regulation, calcium handling, metabolic state, and inhibitory control in the persistence and termination of cramp activity. However, the mechanisms discussed in this review remain incompletely established, and the following considerations should therefore be interpreted as conceptual and hypothesis‐generating rather than definitive therapeutic recommendations.

### 4.1 Targeting the chemical layer: Acid–base balance and calcium regulation

Because alkalosis may influence neuromuscular excitability and calcium handling, careful management of acid–base balance may be relevant in some patients with dialysis‐associated muscle cramps. Potential strategies include optimization of ultrafiltration rates, avoiding excessive fluid removal, and adjusting dialysate bicarbonate concentrations (van der Meulen et al., 1992).

In acute settings, expansion of extracellular fluid volume using isotonic or hypertonic saline may help alleviate cramp symptoms, possibly through effects on ionized calcium availability and extracellular osmotic balance (Takahashi et al., 2002).

Magnesium may also be physiologically relevant because of its modulatory effects on neuromuscular excitability and NMDA receptor activity (Kirkland et al., 2018; Liguori et al., 2024; Souza et al., 2023). However, the specific contribution of magnesium to muscle cramp pathophysiology remains incompletely understood, and further studies are needed to define optimal therapeutic approaches.

Taken together, these interventions may help modulate conditions associated with increased neuromuscular excitability, although their precise mechanisms and clinical efficacy likely vary depending on physiological context.

### 4.2 Targeting the metabolic layer: Energetic support and metabolic flexibility

Because muscle relaxation depends on ATP‐dependent physiological processes, altered energy metabolism may influence the efficiency of contraction termination under metabolically stressed conditions. Carnitine supplementation is widely used in dialysis patients and has been associated with improvement of muscle symptoms in some studies (Sakurauchi et al., 1998; Takahashi, 2021). However, therapeutic responses remain variable, and current evidence does not support a singular causal relationship between carnitine deficiency and muscle cramps.

Accordingly, metabolic interventions may be better understood as approaches aimed at supporting metabolic flexibility and energetic support for ATP‐dependent cellular processes rather than simply restoring ATP concentrations. Adequate levels of water‐soluble vitamins involved in mitochondrial metabolism and oxidative phosphorylation may also contribute to maintenance of normal muscle physiology. In addition, coenzyme Q10 has been investigated for potential effects on mitochondrial function (Ernster & Dallner, 1995; Garrido‐Maraver et al., 2014).

In addition to mitochondrial ATP production, phosphocreatine metabolism represents an important rapid buffering system for maintenance of intracellular ATP during periods of increased energetic demand (Guimarães‐Ferreira, 2014; Saks et al., 1978). Phosphocreatine can rapidly regenerate ATP from ADP through creatine kinase‐mediated phosphate transfer and may therefore contribute to preservation of ATP‐dependent relaxation processes during sustained muscle activation. Because muscle cramps involve high‐frequency motor unit discharge and rapid contractile activity rather than slow aerobic exercise alone (Minetto et al., 2011), disturbances in immediate energetic buffering mechanisms may also influence the persistence and termination of contraction (Dahlstedt et al., 2001).

Taken together, these considerations indicate that a comprehensive metabolic approach—rather than single‐agent supplementation—may be required to effectively restore ATP‐dependent and energetically supported relaxation mechanisms (Nelson & Churilla, 2016).

These considerations suggest that broader metabolic support strategies may influence relaxation efficiency under conditions of metabolic stress, although further investigation is required to clarify their physiological and clinical significance.

### 4.3 Targeting the neural layer: Modulation of excitability and inhibitory control

Current evidence suggests that sustained motor unit activity and altered inhibitory control represent central mechanisms underlying muscle cramps. Accordingly, therapies that modulate neuronal excitability may have physiological relevance in selected patients.

Pharmacological agents such as gabapentinoids may reduce neuronal excitability by modulating calcium channel activity and excitatory neurotransmitter release (Fink et al., 2002).

In addition, spinally acting muscle relaxants such as tizanidine (an α2 receptor agonist) may reduce excitatory polysynaptic spinal transmission and motor neuron excitability through presynaptic inhibitory mechanisms (Davies, 1982) and baclofen (a GABA‐B receptor agonist) may influence inhibitory neural pathways (Capaday, 1995). However, evidence supporting their use specifically in dialysis‐associated muscle cramps remains limited, and caution is required, particularly in patients with impaired renal clearance.

In addition to pharmacological interventions, non‐pharmacological strategies may help modulate neural excitability and facilitate termination of cramp activity. Stretching of the affected muscle may alter force–length relationships of actin–myosin interactions, mechanically disrupt persistent cross‐bridge attachment, and increase inhibitory input from Golgi tendon organs, thereby facilitating termination of sustained motor unit firing (Schwellnus, 2009). Recent evidence suggests that muscle spindle behavior may be influenced not only by muscle length but also by post‐contraction changes in spindle sensitivity and local intramuscular pressure (Torell & Dimitriou, 2024; Wilson et al., 1995). These observations raise the possibility that stretching may help normalize abnormal afferent feedback by modifying both mechanical and sensory conditions within the contracted muscle, although the precise physiological mechanisms remain incompletely understood. Postural adjustments that reduce nerve compression may also be beneficial in selected patients.

### 4.4 Integrated management: A multidimensional physiological perspective

The present framework suggests that muscle cramps may arise through the interaction of neural, chemical, and metabolic factors rather than through a single isolated mechanism. Consequently, interventions targeting only one physiological domain may not fully address the complexity of cramp persistence in some patients.

An integrated clinical approach may therefore include:

- optimization of dialysis‐related physiological conditions,
- support of metabolic and mitochondrial function,
- and modulation of neural excitability and inhibitory control.

Importantly, these concepts should be interpreted as a multidimensional physiological perspective intended to guide future investigation rather than as evidence‐based treatment algorithms. Nevertheless, this integrative framework may help explain variability in symptom presentation and therapeutic response across patients and clinical settings, including dialysis‐associated and exercise‐associated muscle cramps.

## 5 CLINICAL CONTEXTS RELEVANT TO THE INTEGRATED FRAMEWORK

The integrated framework proposed in this review may help contextualize several clinical situations in which muscle cramps are particularly frequent, persistent, or difficult to manage. The following examples are not intended as independent mechanistic explanations, but rather as illustrative clinical contexts in which neural, metabolic, and physiological factors may interact to influence cramp susceptibility and persistence.

### 5.1 Spinal canal stenosis and neural disinhibition

Certain neurological conditions such as spinal canal stenosis may provide clinical examples supporting the importance of altered inhibitory control and sustained motor neuron excitability in cramp persistence (Eliav et al., 2001).

### 5.2 Circadian and metabolic influences on cramp susceptibility

Muscle cramps frequently occur during the night (Rabbitt et al., 2016) or toward the end of dialysis sessions, suggesting that systemic physiological stress and altered neuromuscular excitability may influence cramp susceptibility.

## 6 FUTURE DIRECTIONS

Future research should clarify how neural excitability, inhibitory regulation, calcium handling, and metabolic stress interact across different cramp conditions using integrated electrophysiological and physiological approaches.

## 7 CONCLUSION

Muscle cramps remain a common yet incompletely understood clinical and physiological phenomenon observed across diverse settings, including hemodialysis and exercise. Although previous models have emphasized altered motor neuron excitability, electrolyte imbalance, hemodynamic instability, and metabolic disturbances, no single mechanism fully explains the initiation, persistence, and variability of cramp activity.

In this review, I propose an integrated framework in which muscle cramps may involve impaired termination of contraction resulting from interacting neural, metabolic, and peripheral physiological factors. Within this framework, sustained motor unit activity and altered inhibitory control are recognized as central mechanisms, while altered calcium handling, metabolic constraints, impaired energetic support, and cross‐bridge persistence may contribute to the persistence or inefficient termination of contraction under specific physiological conditions.

Importantly, the concept of “failure of relaxation” presented here is not intended as a singular mechanistic explanation centered solely on ATP depletion or SERCA dysfunction. Rather, it is proposed as an integrative physiological perspective describing conditions in which normal relaxation processes become incomplete or inefficient because of interacting abnormalities in neural regulation, calcium dynamics, and metabolic state.

This framework may help reconcile diverse clinical and experimental observations regarding muscle cramps and may provide a useful conceptual basis for future physiological and translational investigation. At the same time, many aspects of the proposed framework remain speculative, and further research is required to clarify the relative contribution of neural, metabolic, and peripheral mechanisms across different cramp conditions.

Ultimately, muscle cramps may be better understood not simply as disorders of excessive activation alone, but also as conditions in which impaired termination of contraction may contribute to cramp persistence under certain physiological conditions.

## AUTHOR CONTRIBUTIONS

**Akira Takahashi:** Conceptualization; investigation; visualization.

## FUNDING INFORMATION

The author has nothing to report.

## CONFLICT OF INTEREST STATEMENT

The author declares no conflicts of interest.

## ETHICS STATEMENT

This study is a narrative review and does not involve human participants or patient data.

## CONSENT

The authors have nothing to report.

## Data Availability

The author has nothing to report.
